# Translating the Pelvic Organ Prolapse Score into Samoan using a modified back translation methodology

**DOI:** 10.1186/s12905-022-01676-3

**Published:** 2022-03-27

**Authors:** Melanie Dembinsky, Ramona Boodoosingh, Saunima’a Ma Fulu-Aiolupotea, Uila Laifa Lima, Alec Ekeroma, Carol Bugge, Suzanne Hagen

**Affiliations:** 1 Faculty of Health Sciences and Sport, University of Stirling, Stirling, UK; 2 School of Nursing and Health Science, National University of Samoa, Apia, Samoa; 3 Nursing, Midwifery and Allied Health Professions Research Unit, Glasgow Caledonian University, Glasgow, UK

**Keywords:** Pelvic organ prolapse, POP-SS, Samoan translation

## Abstract

**Background:**

Although Samoan women have a high prevalence of obesity and multiple parity which are risk factors of pelvic organ prolapse, there is no prevalence data on this condition.

**Aims:**

Translate the Pelvic Organ Prolapse-Symptoms Score (POP-SS) from English into Samoan,

**Materials and methods:**

Standardised methods for translating questionnaires, individual face to face audio-recorded interviews in which women completed the POP-SS using a Think Aloud method, analysis using a Framework approach.

**Results:**

The POP-SS was successfully translated in to Samoan, an additional information leaflet was developed to support women’s understanding of what prolapse is, 14 Samoan women were recruited of which 13 were interviewed and completed the POP-SS, results of POP-SS (scores), results of think aloud, results in terms of research experience.

**Conclusions:**

A Samoan version of the POP-SS is now available for further evaluation of its psychometric properties prior to wider use. The team continue to collaborate on their work on establishing the prevalence of prolapse whilst building local research capacity.

**Supplementary Information:**

The online version contains supplementary material available at 10.1186/s12905-022-01676-3.

## Background

Pelvic organ prolapse is a common condition affecting, in the Western world, 40% of women over the age of 50 [1]. A considerable amount of research has been conducted examining various aspects of prolapse, its prevalence and treatment [2–5], but data from any of the Pacific Island nations is absent. This is concerning given that Pacific Island women have high levels of two of the key risk factors for prolapse: obesity (rates in the Pacific Islands are among the highest in the world at approximately 70%) and high fertility rates (on average 3.7 births per woman). Because of these high levels of known risk factors, these populations are likely to have high levels of prolapse, yet the prevalence is not known. Additionally, there are commonly undiagnosed genital injuries which increase the risk of prolapse further [6, 7].

Hagen et al. [8] developed the brief 7-item Pelvic Organ Prolapse Symptom Score (POP-SS) to address the lack of a simple symptom score, and have published on its internal consistency, construct validity, test–retest reliability and sensitivity to change [8, 9]. The POP-SS has been widely used, both in the research and clinical contexts, and has been translated into Turkish, Amharic and Chinese and Nepali [10]. The POP-SS, if translated, has the potential to be used in establishing the prevalence of symptomatic prolapse in Pacific Island Nations.

We undertook the current study to: develop a Samoan version of the POP-SS to be used in the future collection of prevalence data in Samoa; provide the first data about this condition in the Pacific Islands; build capacity in Samoa in relation to methods for rigorous translation of questionnaires for the purposes of epidemiological and clinical research; establish transferrable methods for translation of the POP-SS into other Pacific Island languages.

Samoa is a small developing nation located in the South Pacific, with the majority of its estimated population of 198,646 [11] residing on the two major islands of Upolu and Savaii where the government hospitals and district health facilities are based. There is a high prevalence rate of two key risk factors for the development of pelvic organ prolapse, multiple parity and obesity. Although the fertility rate total (births per women) has declined from the 1960 rate of 7.651, it is still high at 3.877 in 2018 [12]. The rate of obesity among women has increased from 44.4% [13] to the current estimate of 81% [14].

## Methods

This research aimed to translate the POP-SS into Samoan using the back translation method, a recognized method of translation [15, 16] and to pilot the translated instrument with a small sample of Samoan women. The study was conducted between March and September 2020 with data collection commencing between June and July 2020 due to COVID-19 restrictions in Samoa.

The back translation process was done by two teams, comprising two individuals each from the School of Nursing and the Centre for Samoan Studies at the NUS. Using this methodology, the teams went through the sequence of translating the POP-SS from English to Samoan, followed by translation of the instrument from Samoan to English, then both teams met to confer and discuss any areas requiring clarification; the process was again repeated.

All translators had previous experience of translating Samoan to English and English to Samoan. This experience had been gained through other research projects to translate various documents such as consent forms, surveys and interview transcripts. The process of back translation was new to the researchers as prior research did not have the translation work as its main objective.

The translation team experienced a number of challenges which were exacerbated by the constraints on movement and limited access to internet due to the COVID-19 state of emergency measures imposed in Samoa in the early part of 2020. These challenges included limited in-person interaction (due to restrictions on movement and access to the campus) with the other team members, which required sending documents via email and phone calls and the feeling of uncertainty on their progress. Once restrictions were eased, researchers met for an in depth in-person final rich spirited discussion on the minutia of the content of the POP-SS. Although disagreements emerged in the process on the form, the use of symbols and wording due to the contextual nature of the language, these were resolved through respectful and active dialogue.

In Samoa, the reproductive health system is considered a sensitive topic and the language health workers use is tempered by the reality that many patients are accompanied to the health facilities, so questions must be asked in a manner that conveys the meaning accurately while maintaining respect for the patient and the person who accompanied them. The nuanced nature of the terms in the POP-SS presented a challenge with regards to balancing the conveyance of the information while being culturally sensitive and appropriate. There are multiple versions of the Samoan language used for formal and informal communication, each with a distinct vocabulary. Formal Samoan is well known to be highly contextual, but is not spoken or understood by all Samoans. As nurses made up part of the translation team they were able to share the terminology and form of Samoan used in the field to communicate with participants, delicately balancing a respectful tone and integrity of the information that was being conveyed.

## Materials

The materials used were the English version of the POP-SS (Additional file 1) which was translated into Samoan (Additional file 2), and a leaflet (Additional file 3) that was presented to interview participants. Half of the participants received the leaflet alongside the Samoan POP-SS, and the other half received the leaflet after completing the Samoan POP-SS. We wanted to examine if the availability of the leaflet alongside the questionnaire would aid Samoan women in understanding the POP-SS given the limited awareness of female anatomy among the population.

## Participants

Parous Samoan women over the age of 18 were eligible for participation. Women who were not of Samoan descent, weren’t fluent in Samoan and had not given birth were excluded. Participation in the study was voluntary and written consent was obtained at the beginning of the interview. The interview package was produced in Samoan and contained an information sheet, consent form, visual material on pelvic organ prolapse (leaflet), additional demographic questions and the Samoan POP-SS. The leaflet showed and explained what different types of prolapse look like, what it is and how it is different to a cyst to enhance clarity between these two. To gauge feedback on the visual materials, half of the participants received the leaflet alongside the translated POP-SS (Group A). The other half was presented with the leaflet after they had commented and completed the Samoan POP-SS (Group B).

Women were invited to participate using the snowball method due to the small sample required and the sensitivity of the topic. The two members of the translation team from the School of Nursing conducted the interviews with the pilot sample of 13 women using the Think Aloud method [17]. As the two researchers had not used this method previously, a member of the UK team did a mock interview with each of them to help with familiarisation of the technique. Participants were asked to think aloud while completing the translated Samoan version of the POP-SS. No physical examination was performed to assess the presence of POP for any of the participants.

Interviews were audio recorded using a small unobtrusive recording device and transcribed into Samoan by the two researchers. The transcripts were then translated from Samoan to English by the researchers conducting the interviews using a contextual approach as well as verbatim translation as some phrases imply emotion which are difficult to translate directly.

## Data analysis

A Thematic framework approach [18] was used by three researchers (MA,RB,MD) for data analysis. Five themes were identified: clarity of the question, feedback about taking part in the study/research, additional information provided in response to the question, reproductive concerns and questions, and response to the visuals provided. MD provided guidance to RB and MA, who had relatively little to moderate experience in thematic analysis. Coding of the transcripts was compared routinely between MD and the Samoan researchers. The coding process also aided the interviewers to understand where they had lapsed in the think aloud method themselves.

Women were asked to complete the Samoan POP-SS and the scores were calculated, by adding the responses for each question. Higher scores indicate the presence of more prolapse symptoms. We had only asked women to complete the questionnaire to explore if the questions were understood, and not to perform a meaningful analysis of the scores as the sample is too small.

## Results

Seven women received the leaflet alongside the translated POP-SS and seven received it after completion of the questionnaire. We had divided the sample into two groups to investigate if the translated version of the POP-SS would be better understood if women were provided with some information about relevant aspects of the female anatomy prior to completing the POPSS. Half of the women were provided with information prior to completing the POPSS and half were provided the information after. As all women indicated that having the leaflet would aid women’s understanding of the POP-SS questions, we present the sample as a total.

One participant provided feedback on the translated POP-SS version and the visual materials (leaflet) but did not complete the questionnaire. The overall sample of the study therefore is 14, with 13 completing the translated version of the POP-SS in addition to providing feedback on clarity and acceptability of the translated version of the POP-SS and the visual materials (leaflet).

Participants’ age ranged from 29–63 with a median age of 35 years, nine lived within Apia and the remaining four in villages located on Upolu. Further demographic characteristics of participants are presented in Table 1.

**Table 1:** Participant characteristics (n = 13)

Age in years	
Mean	38.3
Median (range)	35 (29–63)
SD	8.98
Parity	
Mean	4.2
Median (range)	4 (1–10)
SD	2.28
Residence (n)	
Urban	9
Rural	4
POP-SS score	
Mean	3.38
Median (range)	2 (0–12)
SD	0.87

Data analysis of the transcripts highlighted a few minor errors within the translations which were immediately addressed and rectified. All participants deemed the questions culturally acceptable.

“Reading this paper, it is clear, its statements are well written and clearly worded in the way you have translated the paper into Samoan” (MA06B)

“It is very clear to me, I don’t know because you were the ones that did the translation but the Samoan meaning to me is very clear”. (MA08B)

Feedback from participating women was predominantly in favour of providing the additional information and visuals about pelvic organ prolapse.

“To me, even though these are taboo to our culture and our people may think that we are very disrespectful, but I really want you to use these pictures. Yes, I know this survey and the topic is related to mothers but most of them don’t really know where these organs are located. Most of us also never really see these organs of themselves. This is good, it is clear, and it is easy… to me. […] Those words are appropriate. Believe me, those words are easily understood. There is nothing that means too polite.” (MA10B)

Women reported that this created a better understanding of the questions, but also about their own anatomy which was welcomed by participants as it remains a sensitive subject within Samoa.

“Thank you for this research that I have knowledge that there are different openings as the urethra and the vagina as well. I though there is only one opening where urine comes from and also to have sexual intercourse. And from the diagrams that you have shown made me learned that these are different openings. (UL01A)

“As a person with background I think having additional information will assist a person that has no knowledge in things like the reproductive organs and the problems that has been discussed.” (UL14B)

Participating women frequently praised the development of a Samoan version of the questionnaire and the opportunity to participate in research focused on them as Samoan women. The research and accompanying visual aids enhanced women’s understanding of the female genital anatomy and physiology. It further stimulated discussion on other gynaecological concerns, which participants raised. Several participants had experienced continual discomfort but not sought assistance as it had been perceived as normal and speaking about the genital region is considered embarrassing.

## Discussion

An acceptable Samoan version of the POP-SS has been developed alongside an information leaflet which illustrates the normal location of the pelvic organs compared to displaced ones when experiencing a prolapse. Participants completed the translated POP-SS and the scores ranged from 0–12. Higher scores indicate higher presence of prolapse symptoms and increased bothersomeness to the woman. Within our very small sample of women the scores were low, suggesting that some women had some bothersome prolapse symptoms, but not many of them did. These results need to be considered with extreme caution as the sample was very small, but even within this very limited sample there were women who indicated that they experienced prolapse symptoms, warranting further explorations at a larger scale.

This is the first time the POP-SS has been translated into a Pacific Island country’s language. However, the POP-SS has been translated into Chinese [19], Amharic [20], Nepali [21] and Turkish [22]. Compared to the current study which only completed the translation and pre-testing phase, the other POP-SS translations have been evaluated for reliability and validity. It is the authors’ intention to carry out a full evaluation process for the Samoan version of the POP-SS as the next step prior to its clinical and research use.

Good practice was applied in the selection of the translation teams, who could bring not only “sociolinguistic language competence [23, p. 280] but also were knowledgeable about the subject matter” [24, p. 287]. One part of the translation team for the POP-SS was made up of two registered nurses, who also administered the pilot to the 14 participating women. The other part of the translation team were faculty at the Centre for Samoan Studies who were experienced translators and knowledgeable on Samoan culture and language. The translation teams for the POP-SS, piloting and analysis of the POP-SS interviews were also part of the research team which was beneficial, as they brought “valuable intercultural knowledge to the research process” [24, p. 287].

The nuanced nature of the translation of the POP-SS from English to Samoan was underpinned by the lack of direct translations from one language, a challenge that can exist in the translation of “health care language” [23, p.279]. The Samoan language is rich in context, with a formal and informal form, each appropriate to different situations and audiences [25], which contributed to rich discourse between the translation teams on the form of Samoan that would be most appropriate for the intended audience. Several of the symbols such as ‘/’ used in the English language to represent ‘or’ do not have a direct Samoan language equivalent. This was detected during the pilot interviews, hence the recommendation moving forward to have words spelt out and to remove symbols.

The process used in this translation was forward translation, back translation and a meeting of the two research teams, with the process being repeated. There is some debate on the merits and disadvantages of the back translation process, and in this research the back translation process is modified from the commonly associated stages of forward translation into the new language, back translation into the original language, followed by a review by an expert committee. One argument raised by Behr [26, p. 574] is “the general notion of a forward translation being flawed and a back translation being flawless is inappropriate: Discrepancies when comparing original and back translation can be due to errors in the actual translation but they can also be due to errors in the back translation”.. This research team did not approach the back translation methodology with this thought process, recognizing that in the highly contextual Samoan, it would require open and continuous discussion on the appropriateness of the translation. In addition, the POP-SS has been successfully translated into several other languages using the back translation methodology and tested well with high levels of validity.

In terms of future research, further piloting of the Samoan POP-SS is needed in a larger more diverse sample of women from a number of different settings to assess its acceptability and face validity. Also an assessment of its construct validity, internal consistency and test retest reliability is required. Once there is evidence that the Samoan POP-SS has strong psychometric properties, a prevalence study is planned to estimate how widespread prolapse is amongst Samoan women. This research will be taken forward as part of the Pacific Islands PElvic fLoor dysfunctIon Network (PIPELINE) funded by the Academy of Medical Sciences.

## Conclusion

In conclusion, the adapted back translation methodology yielded promising results in the translation of the pelvic organ prolapse symptom score, and as a low cost, low resource requirement strategy can provide an option for other developing countries to translate standardized health screening tools into local languages.

## Supplementary Information

**Additional file 1**. English POP-SS.

**Additional file 2**. Samoan POP-SS.

**Additional file 3**. Samoan leaflet with prolapse information.

## Data Availability

The anonymous dataset used and analyzed in this study is available from corresponding author on reasonable request. Transcripts of the interviews are not available in their entirety to protect participants’ anonymity.
