# Supplementary material for: Translating the Pelvic Organ Prolapse Score into Samoan using a modified back translation methodology
Source: BMC Womens Health. 2022 Mar 27;22:93. doi: 10.1186/s12905-022-01676-3 (PMC8960078; doi:10.1186/s12905-022-01676-3)
Supplement: Supplementary file 1 — Additional file 1. English POP-SS. [file 12905_2022_1676_MOESM1_ESM.pdf]

Pelvic Organ Prolapse Symptom Score

We would be grateful if you could answer the following questions, thinking about how you have been, on average, over the **PAST FOUR WEEKS**. ***(Please cross one box in each row)***

	**How often during the last four weeks have you had the following symptoms:**	**Never**	**Occasion-ally**	**Some-times**	**Most of the time**	**All of the time**
**A1**	a feeling of something coming down from or in your vagina?	☐ 0	☐ 1	☐ 2	☐ 3	☐ 4
**A2**	an uncomfortable feeling or pain in your vagina which is worse when standing?	☐ 0	☐ 1	☐ 2	☐ 3	☐ 4
**A3**	a heaviness or dragging feeling in your lower abdomen (tummy)?	☐ 0	☐ 1	☐ 2	☐ 3	☐ 4
**A4**	a heaviness or dragging feeling in your lower back?	☐ 0	☐ 1	☐ 2	☐ 3	☐ 4
**A5**	a need to strain (push) to empty your bladder?	☐ 0	☐ 1	☐ 2	☐ 3	☐ 4
**A6**	a feeling that your bladder has not emptied completely?	☐ 0	☐ 1	☐ 2	☐ 3	☐ 4
**A7**	a feeling that your bowel has not emptied completely?	☐ 0	☐ 1	☐ 2	☐ 3	☐ 4

A8 which of the symptoms above (questions A1 to A7) causes you most bother? *Please enter a number from 1 to 7 in the box, or cross "Not applicable"* **A** ☐ Not applicable ☐ 99

Copyright © 2008 S Hagen
