# Supplementary material for: Translating the Pelvic Organ Prolapse Score into Samoan using a modified back translation methodology
Source: BMC Womens Health. 2022 Mar 27;22:93. doi: 10.1186/s12905-022-01676-3 (PMC8960078; doi:10.1186/s12905-022-01676-3)
Supplement: Supplementary file 2 — Additional file 2. Samoan POP-SS. [file 12905_2022_1676_MOESM2_ESM.pdf]

O āuga ole āfaina o Tōtoga ole Suilapalapa

O lenei pepa e fa'aāogaina e fesoasoani ai iā te oe, ile siakiina o āuga ole āfaina o tōtoga ole suilapalapa ma ona suiga mai ilea taimi ilea taimi.

Tali fesili olo'o mulimuli mai, mafaufau pē na fa'apefea mai lou soifua maloloina ile **fā vaiaso talu ai**. (Fa'amolemole fa'ailoga se pusa se tasi i laina ta'itasi).

	E fa'afia ona e maua i āuga nei ile fā vaiaso talu ai:	Leai	Seāseā	Nisi o Taimi	Ile tele o taimi	I taimi uma lava
F1	O se fa'alogona o iai se mea o paū po'o le oso mai i fafo o lou itutino sā?	☐ 0	☐ 1	☐ 2	☐ 3	☐ 4
F2	O se fa'alogona lē manuia pē tigā i lou itutino sā e fa'atuputupulaia pe'ā e tū?	☐ 0	☐ 1	☐ 2	☐ 3	☐ 4
F3	O se fa'alogona mamafa pē totoso ile pito i lalo o lou laualo po'o lou ona?	☐ 0	☐ 1	☐ 2	☐ 3	☐ 4
F4	O se fa'alogona mamafa pē totoso i le pito i lalo o lou tua?	☐ 0	☐ 1	☐ 2	☐ 3	☐ 4
F5	O se fa'alogona po'o se mana'oga e fia oono mai i fafolau feau vai?	☐ 0	☐ 1	☐ 2	☐ 3	☐ 4
F6	. O se fa'alogona e le'o māe'a lelei ona tatala mai lau fe'au vai?	☐ 0	☐ 1	☐ 2	☐ 3	☐ 4
F7	O se fa'alogona e le'o māe'a lelei ona tatala mai lau fe'au mamao?	☐ 0	☐ 1	☐ 2	☐ 3	☐ 4

F8 O fea o āuga olo'o taua i luga (F1-7) olo'o pito i sili ona e āfāina ai? Faamolemole tusi i lalo se numera 1-7 i totonu ole pusa F, pe kolosi le pusa "lē talafeagai".

F ☐ Lē Talafea gai ☐
