# Supplementary material for: Translating the Pelvic Organ Prolapse Score into Samoan using a modified back translation methodology
Source: BMC Womens Health. 2022 Mar 27;22:93. doi: 10.1186/s12905-022-01676-3 (PMC8960078; doi:10.1186/s12905-022-01676-3)
Supplement: Supplementary file 3 — Additional file 3. Samoan leaflet with prolapse information. [file 12905_2022_1676_MOESM3_ESM.pdf]

O le a le pa'ū mai i fafo o tōtōga o le Suilapalapa?

O le pa'ū/oso mai i fafo e mafua mai ona ua alu ese mai le tagāmimi/taga ole feauvai, le alāfe'au mamao po'o le to'ala fanau mai i ō latou nofoaga masani/tumau.

O ata o lo'o i lalo, o lo'o faailoaina ai nofoaga masani/tumau o ia tōtōga, atoa ai ma ituaiga pa'ū/oso eseese mai o nei totoga e tolu.

O le pa'ū/oso mai o ia tōtōga, e ese mai se patu po'o se fula e pei ona fa'ailoaina atu ile ata mulimuli.

Uterus – taga fanau/ to'ala fanau
Rectum – alā fe'au mamao
Bladder – tagāmimi
Urethra – alā fe'au vai
Vagina – itutino sā

Normal position
Nofoaga masani/tumau

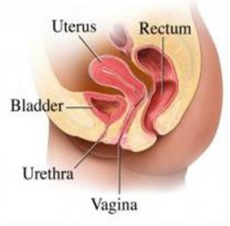

Bladder (front wall) prolapse
Paū o le tagamimi/Tagafeauvai (pito I luma)

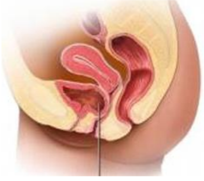

Rectum (back wall) prolapse
Pa'ū o le Alafaeu mamao (pito I tua)

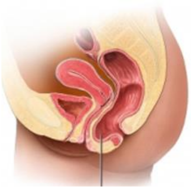

Uterus (womb) prolapse
Pa'ū o le Taga fanau (toala) pito I tua

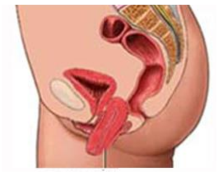

Cyst – NOT prolapse
Fula/Patu – E LE OSE
pa’ū/oso mai

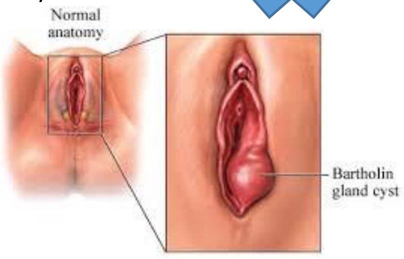

E mafai ona maua uma ia ituaigā pa’ū/oso ise tinā e toatasi ile taimi e tasi pē sili atu fo’i.

O le pa’ū/oso mai o nei tōtōga mai se tinā e mafai ona maua ai ini auga ese’ese.

E tāua tele le silafia po’o ā āuga o a’afia ai se tinā ae pē fa’afia fo’i ona maua ai.

O fesili o lo’o ile laupepa o lo’o soso’o ai, e matou te manana’o ina ia e fa’atumuina mai, ona ose auala lea mo le fa’amauina o nei āuga

Ua ta’ua “Ole faatulagaina/fa’asikoaina o āuga o le Pa’ū mai i fafo o tōtōga o le Suilapalapa”
